# Research on requirements for smart home-based older adult(s) care service platforms based on the Kano model

**DOI:** 10.3389/fpubh.2026.1962040

**Published:** 2026-10-20

**Authors:** Zheng Xianheng, Wang Ling, Zhou Tao

**Affiliations:** 1 Nantong Institute of Technology, Nantong, Jiangsu, China; 2 University of Science and Technology Liaoning, Anshan, Liaoning, China

**Keywords:** age-stratified heterogeneity, better-worse coefficient, contextual design, educational heterogeneity, Kano model, service platform, smart home-based older adult(s) care

## Abstract

**Objective:**

To address the supply–demand mismatch in smart home-based older adult(s) care platforms, overcome the limitations of static Kano classifications, and strengthen sample validity, this study aims to identify the authentic needs of older adults and multi-stakeholder groups.

**Methods:**

Focusing on older adults aged 65 years and above with basic smartphone operational capacity, this study adopted an exploratory mixed-methods design integrating semi-structured interviews with family members and service personnel (*n* = 8) and cross-sectional Kano-model questionnaires (*n* = 506 valid responses from older adults). Sample stability was verified using Chapman-Callegaro criteria; reliability and validity were assessed via Cronbach’s α and the Kaiser-Meyer-Olkin (KMO) test. Berger’s Better-Worse coefficients with Bootstrap 95% confidence intervals (1,000 resamples) were calculated for demand sensitivity grading. Stratified Pearson Chi-square (*χ*^2^) tests were performed across subgroups defined by age, living arrangement (living alone versus co-living with caregivers), and educational level. Parallel simplified Kano questionnaires were distributed to family caregivers (*n* = 58) and front-line service providers (*n* = 41). Nineteen demand items categorized under User (U1–U8), Service (S1–S7), and Environment (E1–E4) dimensions were mapped onto functional, physical, and interaction domains to build a “Creation–Analysis–Application” design model, further validated through back-testing against four real-world deployed platforms.

**Results:**

Across 19 demand items, 8 were classified as Must-be (M), 4 as One-dimensional (O), 5 as Attractive (A), and 2 as Indifferent (I). Social Entertainment (U6) shifted from Attractive (A) toward Indifferent (I) among the 80 + age group (*χ*^2^ = 8.94, *p* < 0.05). Service Status Awareness (U8) was reclassified from One-dimensional (O) to Must-be (M) from the family caregiver perspective. Participants with primary-school education showed higher proportions of Indifferent judgements for complex digital-oriented demands; the 65–70-year-old cohort demonstrated greater prior exposure to digital devices compared with participants older than 70 years.

**Conclusion:**

The improved Kano analytical framework incorporating age-stratified heterogeneity, multi-stakeholder perspectives, living-arrangement differences, and educational-level heterogeneity effectively remedies weaknesses of conventional static Kano classification. Findings provide empirical support for developing graded, user-group-oriented service packages instead of one-size-fits-all feature lists for smart home-based older adult(s)-care platforms.

## 1 Introduction

According to the National Bureau of Statistics, by the end of 2024, China’s population aged 60 years and above reached approximately 310 million (22%), among which people aged 65 years and above exceeded 220 million (15.6%). Projections indicate that the population aged 60 years and above will surpass 400 million by 2035 (1). Influenced by traditional cultural values favoring aging in place, a large proportion of Chinese older adults choose to receive care within their own homes (2). Accordingly, smart home-based older adult(s) care has become a high-priority development direction for digital gerontechnology. The Chinese government has issued a series of policy documents including the Opinions on Promoting the Development of Older adult(s) Care Services (2019) and the 14th 5-Year Plan for National Aging Affairs and the Older adult(s) Care Service System (2022), promoting digital transformation within the older adult(s)-care industry.

Contemporary smart home older adult(s)-care platforms integrate diverse functional modules including safety monitoring, physiological-parameter measurement, daily-life assistance, social-entertainment services, and aging-adaptive interactive interfaces. Safety-oriented functions such as one-click emergency call and automatic fall detection are widely recognized as fundamental requirements for aging in place. Health-monitoring functions enable continuous collection of physiological data; nevertheless, user acceptance varies substantially across age and educational subgroups. By contrast, social-entertainment functions and personalized customisation deliver high satisfaction for the younger-old, yet may lose practical value for the oldest-old experiencing physical frailty and cognitive decline (3). Many existing platforms simply pile up technical functions without differentiated user-group consideration; functions well-suited for one subgroup may bring little practical benefit for others. Under such circumstances, systematic identification of demand attributes from multi-stakeholder perspectives is urgently needed.

Existing literature reveals four major research gaps. First, many demand-acquisition studies rely on small sample sizes without reporting sample-stability criteria or psychometric validation, raising risks of Kano-attribute misclassification. Second, traditional Kano-model applications frequently treat all adults aged ≥65 years as a homogeneous group, neglecting age-related attenuation of attractive-quality demands between younger-old and older-old cohorts (3), as well as educational heterogeneity shaping older adults’ perceptions toward digital services. Third, smart home older adult(s)-care involves multi-party stakeholders including older end-users, family caregivers, and service providers; few studies simultaneously collect Kano evaluations from family and provider standpoints, leading to underestimation of demands such as information transparency. Fourth, most prior research stops at generating demand lists and Kano quadrant outputs, without translating attribute classifications into actionable design logics or validating outputs against real-world deployed platforms. This study fills these gaps through four analytical enhancements: age-stratified subgroup comparison, multi-stakeholder evaluation, Better-Worse coefficient-based sensitivity grading, and real-case backfilling validation. This manuscript constitutes an extension of the first author’s previous master-degree research (4).

## 2 Research content and methods

### 2.1 Theoretical framework

This study rests on two core theoretical foundations: contextual theory and the improved Kano model integrated with Berger’s Better-Worse coefficients.

Contextual theory supports multi-dimensional decomposition of home-based older adult(s)-care demands across three dimensions: User, Service, and Environment. It highlights that user demands cannot be decoupled from real-world usage scenarios; older adults’ physical-cognitive status, service-delivery workflows, and household environmental constraints jointly shape quality-demand attributes (5). In this paper, contextual theory guides the extraction of the 19 demand items and underpins the overall “Creation-Analysis-Application” design model.

The classic Kano model categorizes quality attributes into five types according to relationships between function performance and user satisfaction: Must-be (M), One-dimensional (O), Attractive (A), Indifferent (I), and Reverse (R) (6). To overcome the coarse limitation of quadrant-only judgement, Berger’s Better-Worse coefficients are adopted for quantitative sensitivity assessment (7). The Better coefficient quantifies satisfaction improvement when a function is fully provided; the Worse coefficient quantifies dissatisfaction when the function is absent. Demand-sensitivity value is calculated as *S* = (|Better| + |Worse|)/2, classifying demands into three grades: High (S ≥ 0.75), Medium (0.65 ≤ S < 0.75), and Low (S < 0.65). These sensitivity indicators, later summarised in Table 1, provide objective quantitative evidence for function-priority ranking. Combined with stratified Chi-square testing, this improved Kano framework enables detection of heterogeneous demand differences among older-adult subgroups, family caregivers, and service providers.

**Table 1:** Ranking of older adult(s) care demands and sensitivity grading (authors’ own drawing).

ID	Requirement item	Better	Worse	Cat.	S (Grade)
U1	Daily care	0.509	−0.601	M	0.788 (High)
U2	Health management	0.587	−0.650	O	0.876 (High)
U3	Emergency call	0.401	−0.526	M	0.662 (Med)
U4	Efficient service	0.691	−0.542	O	0.878 (High)
U5	Family contact	0.446	−0.577	M	0.729 (Med)
U6	Social & entertain	0.619	−0.483	A	0.785 (High)
U7	Personalization	0.575	−0.335	A	0.666 (Med)
U8	Service tracking	0.450	−0.581	M	0.735 (Med)
S1	Info security	0.472	−0.501	I	0.689 (Med)
S2	Simple hierarchy	0.518	−0.525	O	0.738 (Med)
S3	Usage tutorial	0.417	−0.394	I	0.574 (Low)
S4	Adaptive UI	0.460	−0.628	M	0.779 (High)
S5	Proactive service	0.574	−0.615	O	0.841 (High)
S6	Multi-sensory IX	0.569	−0.461	A	0.732 (Med)
S7	Positive feedback	0.491	−0.396	I	0.631 (Low)
E1	Safety & privacy	0.454	−0.630	M	0.777 (High)
E2	Env. alerts	0.314	−0.526	M	0.613 (Low)
E3	Self-worth	0.431	−0.601	M	0.740 (Med)
E4	Respect & Underst.	0.584	−0.471	A	0.750 (High)

Cat., category; IX, interaction; Env., environmental. U4 (0.878) and U2 (0.876) are high-sensitivity O-demands, serving as core optimization targets.

### 2.2 Research content

1. Deconstruct three-dimensional demands [User (U1–U8)/Service (S1–S7)/Environment (E1–E4)] for home-based older adult(s) care guided by contextual theory;
2. Apply the improved Kano model and Better-Worse coefficient calculation to quantify demand priority via stratified *χ*^2^ tests and Bootstrap-derived 95% confidence intervals;
3. Construct the “Creation–Analysis–Application” contextual design model and perform backfilling validation against four real-world deployed platforms.

### 2.3 Research methods

An exploratory mixed-methods design was applied. Given that multi-perspective heterogeneous demands for smart home-based older adult(s) care remain insufficiently mapped in existing literature, exploratory mixed-methods are appropriate: qualitative semi-structured interviews first excavate potential latent demands; subsequent quantitative Kano questionnaires validate, classify, and rank these demands. The qualitative phase generates the item pool; the quantitative phase completes demand verification and priority ordering.

Literature review laid theoretical groundwork. Semi-structured interviews were conducted with 8 participants: five adult-child family caregivers and three front-line older adult(s)-care service personnel, to explore latent practical demands. Questionnaires were distributed via two channels: offline paper-based surveys in urban community activity centres and online QR-code questionnaires forwarded by community administrative staff. In total, 562 questionnaires were distributed targeting older adults; 518 questionnaires were returned. After excluding incomplete and invalid responses, 506 valid questionnaires from older adults were retained. Parallel simplified questionnaires were collected from 58 family caregivers and 41 front-line service providers.

Berger’s Better-Worse coefficients (7) were computed to obtain demand-sensitivity S; 95% confidence intervals were generated by Bootstrap resampling (*N*_resample_ = 1,000). All statistical analyses were implemented in SPSS 26.0 with significance level set at *α* = 0.05. This research received ethical approval from the Institutional Review Board of Nantong Institute of Technology (Approval No. NTIT-XSLL-2025-011). All participants provided informed consent prior to participation; all collected data were anonymized for storage and analysis.

## 3 Demand analysis for smart home-based older adult(s) care platforms

### 3.1 Demand acquisition and contextual definition

*Inclusion criteria*: Community-dwelling older adults aged 65 years and above; possessing basic smartphone operational capacity; able to independently understand and complete questionnaire items.

*Exclusion criteria*: (1) Bedridden older adults of any age; (2) individuals suffering from severe cognitive impairment or dementia unable to finish survey tasks; (3) participants with zero smartphone-operation capability.

Older adults experience progressive sensory degradation and cognitive decline, which may contribute to loneliness and technophobia; accordingly, reducing cognitive load represents a core design imperative.

Stakeholders in this service system include older adult end-users, community-based and medical service providers, plus indirect beneficiaries represented by family caregivers. Semi-structured open-ended interviews were carried out with five adult-child family caregivers and three front-line service personnel. After thematic coding, inter-coder reliability reached *κ* = 0.81.

For family-caregiver interviewees, three core thematic concerns emerged. First, high priority was placed on emergency safety: family members expected timely risk alerts when their older relatives encountered danger, corresponding to the requirement of one-click emergency call (U3). Second, family caregivers demanded transparent remote information access: they hoped to inspect service-status updates (U8) as well as blood-pressure and medication-taking records (U2), enabling remote monitoring of their older family members’ conditions. Third, family respondents worried about excessive operational complexity, which would impose extra mental burden both on older users and remote caregivers.

For service-provider interviewees, two dominant themes were identified. On the one hand, front-line staff emphasised service-dispatch efficiency (U4), expecting the platform to push task notifications promptly and reduce redundant manual communication work. On the other hand, they highlighted backend information accessibility: complete user profiles and service-process data should be visible for authorised staff to avoid information asymmetry, which underpins the demand for service-information awareness (U8).

Interview-derived themes were further abstracted and refined. Guided by contextual theory, 19 initial demand items were defined across User (U1–U8), Service (S1–S7), and Environment (E1–E4) dimensions, covering daily care, health management, emergency response, and interface adaptation scenarios. These 19 items constituted the item pool for the formal Kano questionnaire.

### 3.2 Quantitative analysis of demands based on the Kano model

The Kano model, proposed by Kano et al. (6), distinguishes five demand categories: Must-be (M), One-dimensional (O), Attractive (A), Indifferent (I), and Reverse (R). Dual-form (positive- and negative-stem) Kano questions were designed for each of the 19 demand items.

#### 3.2.1 Sample size justification and data results

In total, 518 questionnaires were returned. After filtering invalid and out-of-scope responses, 506 valid samples were kept. Sample-size sufficiency was assessed referencing the “10-times rule” for multivariate analysis (19 × 10 = 190) (8) and Kendall’s heuristic criterion (95–190 samples). Combined with Kano-model stability simulation criteria (reproducibility >80% when *N* ≥ 200) (9), the present sample of 506 meets statistical-stability requirements. A pilot study (*n* = 60) yielded Cronbach’s *α* = 0.86 for positive-form items and *α* = 0.90 for negative-form items; KMO = 0.83 and Bartlett’s test of sphericity was significant (*p* < 0.001), supporting satisfactory reliability and validity. Sample demographic distribution is shown in Table 2.

**Table 2:** Distribution of questionnaire respondents (authors’ own drawing).

Item	Category	Frequency	Percentage (%)
Gender	Male	257	50.79
	Female	249	49.21
Age	65–70 years	272	53.75
	71–80 years	207	40.91
	80 + years	27	5.34
Education	Primary school	38	7.51
	Junior high school	224	44.27
	High school/vocational	62	12.25
	College/Bachelor+	182	35.97

#### 3.2.2 Stratified difference tests

For the full sample, the 19 demands included 7 Must-be (M), 4 One-dimensional (O), 6 Attractive (A), and 2 Indifferent (I) attributes. Pearson *χ*^2^ tests were performed to detect distribution differences across subgroups stratified by age (65–69/70–79/80 + years), living arrangement (living alone / co-living with caregivers), and educational level.

Consistent with existing research evidence, the younger-old group aged 65–70 years generally had greater prior exposure to computers and mobile digital devices compared with participants older than 70 years. Subgroup Chi-square tests for educational background revealed that participants with only primary-school education showed significantly higher proportions of Indifferent (I) evaluations toward complex digital-oriented demands. Educational level acts as an important moderator shaping older adults’ perceptions of platform demands, alongside age and living arrangement.

Further stratified analysis regarding living arrangement uncovered meaningful demand heterogeneity. Among participants living alone, emergency-call function (U3) and environmental-risk alerts (E2) exhibited substantially higher proportions of Must-be (M) assignments. Without on-site family caregivers, solitary-living older adults regard safety-guarantee functions as non-negotiable baseline requirements. By contrast, older adults co-residing with family caregivers assigned higher Attractive-quality weight to social-entertainment (U6) and personalised-service (U7); they tended to pursue experiential improvement beyond pure safety needs.

Key results are presented in Table 3.

**Table 3:** Kano questionnaire results (authors’ own drawing).

Dim.	ID	Requirement name	(A)%	(M)%	(O)%	(I)%	(R)%	*χ* ^2^	Attr.
U	U1	Daily care	28.97	38.15	21.97	10.91	0.00		M
U	U2	Health management	20.23	26.59	38.44	14.74	0.00	5.12*	O
U	U3	Emergency call	27.16	39.60	12.72	19.94	0.58	11.27**	M
U	U4	Efficient service	34.68	19.83	34.22	10.98	0.00		O
U	U5	Family contact	12.72	25.72	31.79	29.48	0.00	7.94*	O → M
U	U6	Social entertainment	40.75	27.17	20.98	10.81	0.29	8.94*	A
U	U7	Personalization	33.53	9.54	23.98	32.95	0.00		A
U	U8	Service status awareness	12.67	25.77	32.19	29.08	0.29	9.63**	O → M
S	S1	Info security	24.28	27.17	22.54	25.14	0.00		M → I
S	S2	Simple hierarchy	37.31	38.01	14.38	10.01	0.29	5.45*	M
S	S3	Usage tutorial	29.77	27.46	11.85	30.61	0.00		I
S	S4	Adaptive UI	20.04	36.71	25.73	16.94	0.58	6.18*	M
S	S5	Proactive service	31.36	35.41	25.72	6.93	0.58		M → O
S	S6	Multi-sensory IX	35.54	24.67	21.39	18.40	0.00		A
S	S7	Positive feedback	38.44	28.92	10.67	21.97	0.00		A → I
E	E1	Security & privacy	41.04	23.41	21.97	13.58	0.00	10.85**	A → M
E	E2	Env. alerts	12.72	33.82	18.50	34.39	0.00	6.52*	I → M
E	E3	Self-worth	18.50	35.55	24.57	21.38	0.00	5.78*	M
E	E4	Respect & Underst.	43.93	32.66	14.16	8.67	0.29		A

A, attractive; M, must-be; O, one-dimensional; I, indifferent; R, Reverse. *p < 0.05, **p < 0.01. “→” indicates reclassification after stratified/multi-role comparison.

Stratified *χ*^2^ tests yielded three key findings:

1. Emergency Call (U3) showed significantly higher Must-be prevalence among the 80 + living-alone subgroup (52.3%) compared with the 65–69 co-living subgroup (31.4%) (*χ*^2^ = 11.27, *p* < 0.01), demonstrating intensified safety demands among the oldest-old.
2. Social Entertainment (U6) decreased from 40.75% Attractive (A) in the 65–69 group to 18.2% Attractive (A), accompanied by increased Indifferent responses (34.5%) within the 80 + group (*χ*^2^ = 8.94, *p* < 0.05), verifying age-related attenuation of attractive-quality demands.
3. Security & Privacy (E1) was significantly re-classified toward Must-be (M) for participants aged 80 + years (*χ*^2^ = 10.85, *p* < 0.01), reflecting rigid privacy-protection demands in this advanced-age subgroup.

#### 3.2.3 Better-worse sensitivity grading

Berger’s Better-Worse coefficients (7) were calculated; 95% confidence intervals were obtained by Bootstrap resampling (1,000 iterations). A two-dimensional quadrant plot was generated using sample-mean Better = 0.513 and sample-mean Worse = −0.519 as axes origins. Demand-sensitivity *S* = (|Better| + |Worse|)/2 was computed to assign each item into High, Medium, or Low-sensitivity grades (Table 1).

Results showed that Efficient Service (U4, *S* = 0.878) and Health Management (U2, *S* = 0.876) were high-sensitivity One-dimensional (O) demands and constituted core optimisation priorities. Emergency Call (U3, *S* = 0.662) and Personalization (U7, *S* = 0.666) fell within the medium-sensitivity tier and formed fundamental service components. Even though Usage Tutorial (S3, *S* = 0.574) was classified as Indifferent (I), it should still be retained as infrastructural support considering the prevailing digital divide among older-adult populations.

#### 3.2.4 Multi-stakeholder comparative analysis

Simplified Kano questionnaires were administered to external stakeholders: family caregivers (*n* = 58) and service providers (*n* = 41). Service Status Awareness (U8) shifted from One-dimensional (O) from older users’ perspective to Must-be (M) from family-caregiver evaluations (Better = 0.61; Worse = −0.58). This finding demonstrates that information transparency is not merely an optional extra feature but a contractual-level requirement essential for sustaining family trust in smart home care services.

### 3.3 Mapping demand attributes to design strategies

Must-be (M): Safety Baseline. Eight items constitute the non-negotiable foundation of the platform. In addition to Emergency Call (U3) and Security & Privacy (E1), Service Status Awareness (U8) and Env. Alerts (E2) belong to Must-be attributes. Platforms should implement proactive exception-alert mechanisms integrating outdoor environmental data (e.g., air quality, road-condition warnings) within baseline monitoring workflows. Adaptive UI (S4) and Simple Hierarchy (S2) form the interaction foundation; complex advanced functions must never undermine basic usability.

From an affordability perspective, Must-be baseline functions should adopt mature, low-cost hardware and software solutions to constrain overall service expenditure. High-cost Attractive-type functions are not mandatory for all user groups and can be implemented as optional modular add-ons. This modular design principle supports the graded-service-package philosophy proposed in this study.

One-dimensional (O): Efficiency & Health. Four items show linear positive correlations between function quality and user satisfaction. Health Management (U2) and Efficient Service (U4) directly drive willingness-to-pay. Platforms ought to integrate IoT-device data with backend service-dispatch systems to shorten the “monitor-alert-response” workflow loops. Proactive Service (S5) exhibited One-dimensional characteristics among digitally literate users, suggesting adoption of progressive-disclosure interaction strategies.

Attractive (A): Emotional Value. Five items contribute to competitive advantage. Social Entertainment (U6) tends toward Indifferent (I) for the 80 + subgroup, reflecting a mismatch between youthful-oriented social functions and the frailty-dominated needs of the oldest-old. Designers should avoid forcing social-function access onto all users; greater emphasis should be placed on Respect & Understanding(E4, e.g., large-font positive prompts, voice-based encouragement) and Multi-sensory IX (S6).

Indifferent (I) & Special Cases. Although Usage Tutorial (S3) and Info Security (S1) obtained Indifferent (I) classification, they cannot be omitted in practical deployment. Usage guidance (S3) should evolve from mandatory pop-up tutorials toward context-triggered onboarding. Identity security (S1) should move away from complex password entry toward biometric authentication plus authorised-proxy management modes.

## 4 Construction of the smart home-based older adult(s) care platform design model

### 4.1 System framework

Drawing upon the “Acquisition-Processing-Application” workflow from contextual theory, the proposed model defines three sequential phases:

1. Context creation: Gather raw multi-dimensional demands across User, Service, and Environment dimensions, define scenario constraints and boundary conditions.
2. Context analysis: Quantify demand attributes via the Kano-Better-Worse analytical framework; map high-priority demands onto interaction, physical, and functional domains to generate concrete design opportunities.
3. Context application: Output prototype specifications and actionable service-system schemes for practical implementation.

### 4.2 Model construction and case backfilling validation

The “Creation–Analysis–Application” model was constructed and retrospectively validated against four real-world deployed smart older adult(s)-care cases:

1. Wuchang District “Smart Older adult(s) Care Service Network” (government-led; G/B/C multi-tiered accounts plus QR-code closed-loop workflow) (10);
2. Shijingshan “Guoyuantong” inclusive care platform (voice ordering with telephone fallback for low-digital-literacy users) (11);
3. Shichahai “Xidao” system (technology-oriented, adopting millimetre-wave radar fall-detection hardware) (12);
4. Ziyang “Smart Health & Care” integrated medical-nursing platform (mini-program-based meal-assistance plus subsidy-card management) (13).

Model-case item-alignment reached 89.5% (17 out of 19 demand items). All four real-world cases prioritised Emergency Call (U3), Health Management (U2), and Adaptive UI (S4). Two notable deviations were observed: Usage Tutorial (S3) was realised via family-proxy assistance in practice (our sample excluded participants with zero digital literacy); Env. Alerts (E2) was merged into indoor environmental monitoring within the Xidao case. Accordingly, the model was refined: S3 guidance was redefined as implicit on-demand guidance; E2 environmental alerts were subsumed into broader environmental-perception modules. This highlights the necessity of gradient adaptive design tailored to older adults’ varying digital-literacy levels.

## 5 Conclusion

1. Demands manifest significant age-stratified heterogeneity. The 65–69 younger-old subgroup prioritises health-related and social-entertainment demands (U6 as Attractive). By contrast, the 80 + oldest-old subgroup prioritises safety and information transparency (U3/U8 as Must-be; U6 shifts toward Indifferent) (*χ*^2^ = 8.94, *p* < 0.05). One-size-fits-all solutions should be replaced by graded service packages. Educational level acts as an important moderator of demand perception: older adults with low educational attainment are more likely to treat complex digital functions as Indifferent items. Living arrangement also produces demand heterogeneity: solitary-living older adults attach higher importance to safety baseline functions, while co-living older adults show stronger preference for experience-enhancing Attractive-type features.
2. Multi-stakeholder Kano comparison shows Service Status Awareness (U8) is re-classified to Must-be (M) within family-caregiver evaluations (Better = 0.61; Worse = −0.58). Information transparency should be treated as a contractual-level baseline requirement rather than an optional add-on function.
3. Better-Worse coefficient-based sensitivity grading provides more fine-grained priority information than simple Kano quadrant classification. Efficient Service (U4, *S* = 0.878) and Health Management (U2, *S* = 0.876) represent high-sensitivity functional-domain optimisation targets.
4. The proposed “Creation–Analysis–Application” contextual-design framework demonstrated good cross-scenario stability (17/19 item alignment) during real-case backfilling validation.
5. Limitations: The 506 older-adult sample was restricted to urban community-dwelling participants; rural-dwelling older adults and severely disabled groups were not included. Sample sizes for family-caregiver (*n* = 58) and service-provider (*n* = 41) subgroups were relatively modest. Our questionnaire battery did not contain items measuring affordability and users’ willingness-to-pay, precluding quantitative analysis of cost–benefit trade-offs.

Future research should adopt multi-city designs incorporating randomised controlled A-B interface-testing. Dedicated continuous technical-support mechanisms should be embedded into smart home older adult(s)-care platform architecture. As highlighted in relevant pre-print work (14), smart home deployment involves not only matching user needs with functional features but also addressing technical support and feedback mechanisms (15). Accordingly, dedicated technical-support mechanisms, technical-team feedback loops, fault-reporting entry points, and remote-assistance channels should be integrated into smart home older adult(s)-care service-system design. In addition, follow-up investigations should add survey items measuring affordability and willingness-to-pay, so as to generate quantitative empirical evidence supporting cost-effective graded service-package configuration (16).

## Data Availability

The raw data supporting the conclusions of this article will be made available by the authors, without undue reservation.

## Appendix. Practical implementation notes for high-priority requirements

Adaptive UI (S4) is identified as a Must-be requirement. Nevertheless, fully personalised multi-branch adaptive interfaces impose substantial debugging, testing, and maintenance burdens for software developers. Unlimited independent interface versions will greatly increase fault-diagnosis complexity.

Rather than building one-to-one fully personalised interfaces for each end-user, this study recommends three-level progressive adaptive configuration: default mode, senior simplified mode, highly-simplified mode. This compromise balances older adults’ usability demands and engineering maintainability.

Additional practical notes for high-priority items:

1. Emergency Call (U3): incorporate an offline fallback mechanism for network-failure scenarios.
2. Security & Privacy (E1): avoid excessive permission collection under data-protection regulations.
3. Social Entertainment (U6): implement user-controllable on–off toggle switches to accommodate heterogeneous preferences across age subgroups.
